# Semen: A modulator of female genital tract inflammation and a vector for HIV‐1 transmission

**DOI:** 10.1111/aji.13478

**Published:** 2021-06-16

**Authors:** Janine Jewanraj, Sinaye Ngcapu, Lenine J. P. Liebenberg

**Affiliations:** ^1^ Centre for the AIDS Programme of Research in South Africa (CAPRISA) Durban South Africa; ^2^ Department of Medical Microbiology University of KwaZulu‐Natal Durban South Africa

**Keywords:** cytokines, epithelial barrier integrity, female genital inflammation, HIV risk, immune cells, semen, vaginal microbiome

## Abstract

In order to establish productive infection in women, HIV must transverse the vaginal epithelium and gain access to local target cells. Genital inflammation contributes to the availability of HIV susceptible cells at the female genital mucosa and is associated with higher HIV transmission rates in women. Factors that contribute to genital inflammation may subsequently increase the risk of HIV infection in women. Semen is a highly immunomodulatory fluid containing several bioactive molecules with the potential to influence inflammation and immune activation at the female genital tract. In addition to its role as a vector for HIV transmission, semen induces profound mucosal changes to prime the female reproductive tract for conception. Still, most studies of mucosal immunity are conducted in the absence of semen or without considering its immune impact on the female genital tract. This review discusses the various mechanisms by which semen exposure may influence female genital inflammation and highlights the importance of routine screening for semen biomarkers in vaginal specimens to account for its impact on genital inflammation.

## 1 INTRODUCTION

Despite the advances made in the treatment of human immunodeficiency virus (HIV), the global HIV prevalence remains unacceptably high.^1^ The primary determinants of HIV transmission include the accessibility of target cells for infection and viral characteristics such as quantity and fitness. Female genital inflammation contributes to both the availability of HIV target cells and reduced mucosal barrier integrity.^2^, ^3^ Genital inflammation, defined by elevated pro‐inflammatory and chemotactic cytokines, has also been linked to a three‐fold greater risk of acquiring HIV in women.^2^ Additionally, microbial dysbiosis contributes to inflammation through increased cytokine production, mucosal barrier disruption and immune cell recruitment at the female genital tract (FGT).^4^, ^5^, ^6^, ^7^ These studies emphasise the role of genital inflammation in HIV acquisition in women and highlight the need to determine factors that contribute to genital inflammation and then limit their relative impact on HIV risk.

The immune altering capacity of semen is often overlooked in heterosexual HIV transmission and semen is merely considered a vehicle for viral transmission to women during condomless sex.^8^, ^9^ Semen induces mucosal changes at the FGT to increase the chances of pregnancy,^10^, ^11^, ^12^, ^13^, ^14^ and also contains several immunologically active molecules known to both promote and inhibit female genital inflammation.^10^, ^11^, ^12^, ^13^, ^15^, ^16^, ^17^, ^18^, ^19^, ^20^, ^21^, ^22^ Initially, the presence of semen in the female reproductive tract results in an inflammatory response involving cytokine production and leukocyte recruitment for the removal of excess and abnormal sperm.^10^, ^11^, ^20^, ^21^ The alkaline pH of semen and the microbial content of the ejaculate also contribute to alterations in the vaginal microbiome which are known to promote genital inflammation and HIV risk in women.^4^, ^5^, ^7^, ^23^, ^24^, ^25^, ^26^, ^27^, ^28^ A semen‐induced pro‐inflammatory immune response to prime the female reproductive tract for conception may also promote genital inflammation and HIV acquisition in women.^11^, ^20^, ^21^, ^22^

Conversely, semen also contains factors to help regulate this pro‐inflammatory response at the FGT since excessive inflammation may lead to adverse pregnancy outcomes. This results in the induction of a regulatory T‐cell (Treg) immune response for tolerance to the paternal antigens and to facilitate embryo implantation.^16^, ^29^, ^30^, ^31^, ^32^ A semen‐induced tolerogenic immune response may also inhibit the clearance of HIV and other pathogens at the FGT. Taken together, these studies suggest that semen directly alters the biology of the FGT and may have significant consequences for the risk of HIV infection in women. Here, we review the relationship between female genital immunity and male partner semen and its implications for HIV risk in women.

## 2 HOST IMMUNE DEFENCES TO PREVENT HIV INFECTION AT THE FEMALE GENITAL MUCOSA

### 2.1 Innate immune responses at the female genital mucosa

#### 2.1.1 Role of the vaginal epithelium in innate immune defence

During male to female HIV‐1 transmission, viral particles present in semen must transverse the vaginal mucus and epithelium to access local cellular targets for infection. However, the FGT has several innate and adaptive immune responses that defend against HIV infection. The innate immune system involves a rapid and non‐specific immune response to injury and infection. Tissue‐associated phagocytes and intact epithelial barriers are among the primary host defences that serve as physical and chemical barriers against HIV infection.^33^ During coitus, semen is deposited in the lower FGT, consisting of the ectocervix and vagina. The lower FGT is lined with several layers of stratified squamous epithelial cells.^34^, ^35^ These cells are held together by tight and adherens junctions, which reduce the permeability of the epithelium and prevent viral entry at the lower FGT.^35^, ^36^, ^37^ Furthermore, the lower FGT has superficial layers of vaginal epithelium consisting of cornified epithelial cells that provide an additional layer of protection.^38^ The upper FGT includes the fallopian tubes and ovaries, uterus, and the endocervix, each lined with a single layer of columnar epithelial cells held together by tight junctions. Vaginal epithelium thickness is influenced by sex hormone fluctuations during the menstrual cycle phases and with hormonal contraceptive use.^39^, ^40^, ^41^, ^42^ Increased progesterone has been associated with epithelial thinning at the FGT and a greater risk of HIV infection.^41^, ^42^, ^43^, ^44^, ^45^ Tissue‐associated phagocytes such as neutrophils engulf and destroy invading pathogens and infected cells through various mechanisms.^33^, ^46^ Neutrophils can release their deoxyribonucleic acid (DNA) to form neutrophil extracellular traps that prevent HIV infection through viral inactivation.^46^ In addition, epithelial and innate immune cells produce cytokines and induce leukocyte recruitment in response to infection.^33^, ^47^

#### 2.1.2 Role of the cervicovaginal mucus in innate immune defence

The cervicovaginal environment is covered in a thick layer of mucus that provides lubrication during coitus, facilitates sperm migration, and acts as a physical and chemical barrier to prevent access to the underlying epithelium.^48^, ^49^, ^50^, ^51^, ^52^ Cervicovaginal mucus (CVM) is primarily composed of water and mucin glycoproteins but also contains immunoglobulin (Ig)G, IgA and several antimicrobial agents which provide additional protection at the female genital mucosa.^49^, ^50^, ^53^, ^54^, ^55^, ^56^, ^57^ The lower FGT is populated by commensal microbes that can modify the CVM composition and influence its ability to defend against pathogens. Acidic CVM associated with *Lactobacillus crispatus* dominance and high levels of D‐lactic acid can hinder HIV‐1 mobility and prevent infection.^52^, ^58^, ^59^ Conversely, HIV mobility is significantly increased in CVM derived from women with bacterial vaginosis (BV).^60^ This is likely since *Gardnerella vaginalis*, a common BV‐associated microbe secretes sialidase enzymes that degrade the CVM.^61^ These findings highlight the complex interplay between the vaginal microbiome and host innate immunity.

#### 2.1.3 Role of the vaginal microbiome in innate immune defence

An optimal vaginal microbiome is dominated by Lactobacilli spp., which exists in a mutualistic relationship with the host and contributes to the immune defences at the FGT.^62^ Commensal microorganisms such as *L. crispatus* prevent pathogen colonisation by inhibiting their growth, preventing biofilm formation, lowering the vaginal pH, competing for nutrients and adherence to the epithelium, and by producing antimicrobial agents such as lactic acid, hydrogen peroxide (H_2_O_2_) and bacteriocin.^63^, ^64^, ^65^, ^66^, ^67^ Lactobacilli metabolise glycogen secreted by vaginal epithelial cells to produce L‐ and D‐isomers of lactic acid.^67^, ^68^ Physiological concentrations of vaginal lactic acid are sufficient to inactivate BV‐associated microbes and other sexually transmitted agents of infection, including HIV.^58^, ^59^, ^69^, ^70^, ^71^ Lactic acid lowers the vaginal pH, enhances the activity of other antimicrobial factors and upregulates the production of anti‐inflammatory cytokines.^67^, ^72^ Taken together, these data suggest that a Lactobacillus‐dominant vaginal microbiome is highly beneficial and less vulnerable to HIV infection.

### 2.2 Adaptive immune responses at the female genital mucosa

Adaptive immunity at the FGT involves either cell‐mediated or humoral immunity. Cell‐mediated immunity involves the removal and destruction of intracellular pathogens and virus‐infected cells by T lymphocytes. Antigen‐presenting cells process and display antigens to T cells to trigger a pathogen‐specific immune response and promote immunological memory. This adaptive immune response is characterised by the involvement of various CD4+ T cell (eg, T‐helper [Th]1, Th2, Treg, T follicular helper [Tfh] and Th17 cells) and CD8+ T cell subsets. Cytotoxic T cells (CD8+) recognise antigens presented on major histocompatibility complex (MHC) class I molecules and directly kill virus‐infected cells by inducing apoptosis through perforin and granzymes.^73^ Conversely, CD4+ T cells recognise antigens presented on MHC class II molecules and respond by secreting cytokines to activate CD8+ T cells, macrophages, and B cells to destroy infected cells.^74^, ^75^

Humoral immunity is mediated by B cells and their secreted antibody products. Antibodies prevent and fight infections by binding to antigens on the pathogen and preventing their entry into host cells, coating the pathogen for phagocytosis, inducing antibody‐dependent cell‐mediated cytotoxicity, and by activating the complement pathway.^76^, ^77^ IgG is the predominant immunoglobulin isotype found in genital secretions of both HIV‐infected and uninfected women.^78^, ^79^ T‐cell immunity and the abundance of immunoglobulins at the FGT are highly regulated by sex hormones.^73^, ^80^

One to two weeks after infection, effector CD4+ and CD8+ T cells die, leaving behind antigen‐specific memory T cells that persist long after infection. Memory T cells mount a rapid immune response upon reinfection with the same pathogen and can be subdivided into central memory cells that circulate between the blood and lymph nodes, and resident and recirculating effector memory cells in non‐lymphoid tissue.^75^, ^81^, ^82^ Tissue‐resident memory T cells (TRMs) reside in mucosal tissues and rapidly respond to local infections by producing cytokines to induce immune cell activation and recruitment at the FGT.^75^, ^83^, ^84^, ^85^ Although the physiological role of TRMs is to defend against infections, these cells have also been identified as major targets for HIV at the lower FGT.^86^, ^87^

## 3 GENITAL INFLAMMATION INCREASES HIV ACQUISITION RISK IN WOMEN

Although the female genital mucosa has several defences to prevent infection and the probability of heterosexual HIV transmission is relatively low, ^9^, ^88^ inflammation can increase the risk of HIV acquisition at this site. This is supported by observations of infection by less fit HIV variants in women with genital inflammation than without.^89^ Inflammation is the body's natural response to injury or infection and involves the influx of immune cells and their products to the site of infection. However, inflammation also contributes to the availability of HIV susceptible cells at the female genital mucosa. Masson et al^2^ demonstrated that genital inflammation, characterised by elevated concentrations in at least 5 of 9 pro‐inflammatory cytokines, was associated with a greater risk of HIV infection in South African women. The study also identified specific cytokines (macrophage inflammatory protein [MIP]‐1α, MIP‐1β, and interferon gamma‐induced protein [IP]‐10] that were independently associated with HIV seroconversion.^2^ The chemokines MIP‐1α, MIP‐1β and IP‐10 are involved in recruiting HIV target cells to the female genital mucosa.^90^, ^91^, ^92^, ^93^ Additionally, elevated cervicovaginal cytokines also contribute to HIV risk in women through mucosal barrier disruption.^3^, ^94^

A compromised vaginal epithelium facilitates HIV entry and access to local immune cells for infection. Elevated pro‐inflammatory cervicovaginal cytokines have been associated with several proteins involved in protease activity, epithelial barrier function, tissue remodelling, and actin cytoskeleton organisation.^3^ Arnold et al^3^ also demonstrated that increased concentrations of matrix metalloproteinases (MMP)‐8 and 9, proteins involved in the remodelling of the extracellular matrix, are associated with raised cytokine biomarkers of inflammation. Elevated levels of MMPs in vaginal fluid from women with BV were also shown to disrupt endocervical epithelial polarisation and increase HIV transmigration through the endocervical epithelium.^6^ Additionally, a study conducted in mice demonstrated that tissue inflammation induced remodelling of the extracellular matrix and altered CD4+ T cell motility.^95^ Tissue remodelling and degradation may result in reduced epithelial barrier integrity thereby facilitating access to HIV target cells at the FGT. Consistent with this, studies have demonstrated an increased risk of HIV infection in women with reduced epithelial barrier function.^96^, ^97^, ^98^ A compromised epithelial barrier may also facilitate microbial translocation^6^, ^94^ and vaginal microbial diversity known to increase HIV infection rates in women.^4^, ^5^, ^7^

Although a lactobacillus‐dominant vaginal microbiome is beneficial to host immunity, South African women tend to have greater microbial diversity.^4^, ^5^ Microbial diversity and BV are linked to an increased risk of HIV infection in women^4^, ^5^, ^7^ and higher rates of both sexual and vertical HIV transmission.^99^, ^100^ Specific BV‐associated bacteria (*Prevotella*, *G. vaginalis*, *Sneathia*, *Parvimonas* and *Gemella*) have been significantly associated with genital inflammation and an increased risk of HIV acquisition in women.^4^, ^5^, ^7^, ^101^ These microbes contribute to inflammation through activation of the nuclear factor kappa B (NF‐κB) pathway, increasing genital cytokines, immune cell recruitment, reduced epithelial barrier integrity, and impaired wound healing.^4^, ^6^, ^49^, ^102^ These studies highlight the role of genital inflammation in susceptibility to HIV infection in women. A better understanding of factors that modulate genital inflammation is required to prevent HIV transmission in women at high risk of acquiring the virus. Here, considering that HIV is predominantly transmitted to women via heterosexual transmission, we review the potential for semen exposure and condomless sex to foster the genital immune environment linked to HIV risk in women.

## 4 THE STRUCTURE OF THE MALE GENITAL TRACT AND HIV INFECTION

The male genital tract (MGT) is comprised of the penile urethra and the testes (Figure ). In uncircumcised males, the foreskin provides both physical and immunological protection to the glans^103^ but is also highly susceptible to HIV infection.^104^, ^105^ The outer surface of the foreskin is lined by a double layer of keratinised stratified squamous epithelium that covers the glans/corona and the opening of the penile urethra (meatus).^104^, ^106^ The epithelium of the foreskin is relatively resistant to HIV infection unless microabrasions are induced during condomless sex, which may facilitate access to target cells within the underlying epithelium.^104^, ^106^, ^107^ The subpreputial cavity, which is the inside of the foreskin, provides an anoxic and moist microenvironment that harbours a diverse array of anaerobic microbes.^27^, ^108^, ^109^, ^110^ The presence of these anaerobic microbes increases the susceptibility of the neighbouring epithelium and the urethral opening to HIV infection via activation of target cells.^108^, ^109^, ^110^, ^111^, ^112^, ^113^ Additionally, when the penis is erect, the foreskin retracts, exposing the glans and inner foreskin, which are more susceptible to viral infection.^114^ The inner foreskin contains HIV target cells that are directly exposed to the vagina during sexual intercourse.^105^, ^114^, ^115^, ^116^, ^117^, ^118^ Medical male circumcision involves the surgical removal of the foreskin resulting in a dry keratinised epithelial surface that is more resistant to HIV infection.^119^, ^120^, ^121^ Circumcision also reduces the diversity of the penile microbiota and may decrease HIV acquisition risk in both men and women.^108^, ^122^, ^123^, ^124^, ^125^, ^126^, ^127^, ^128^

**FIGURE 1:**
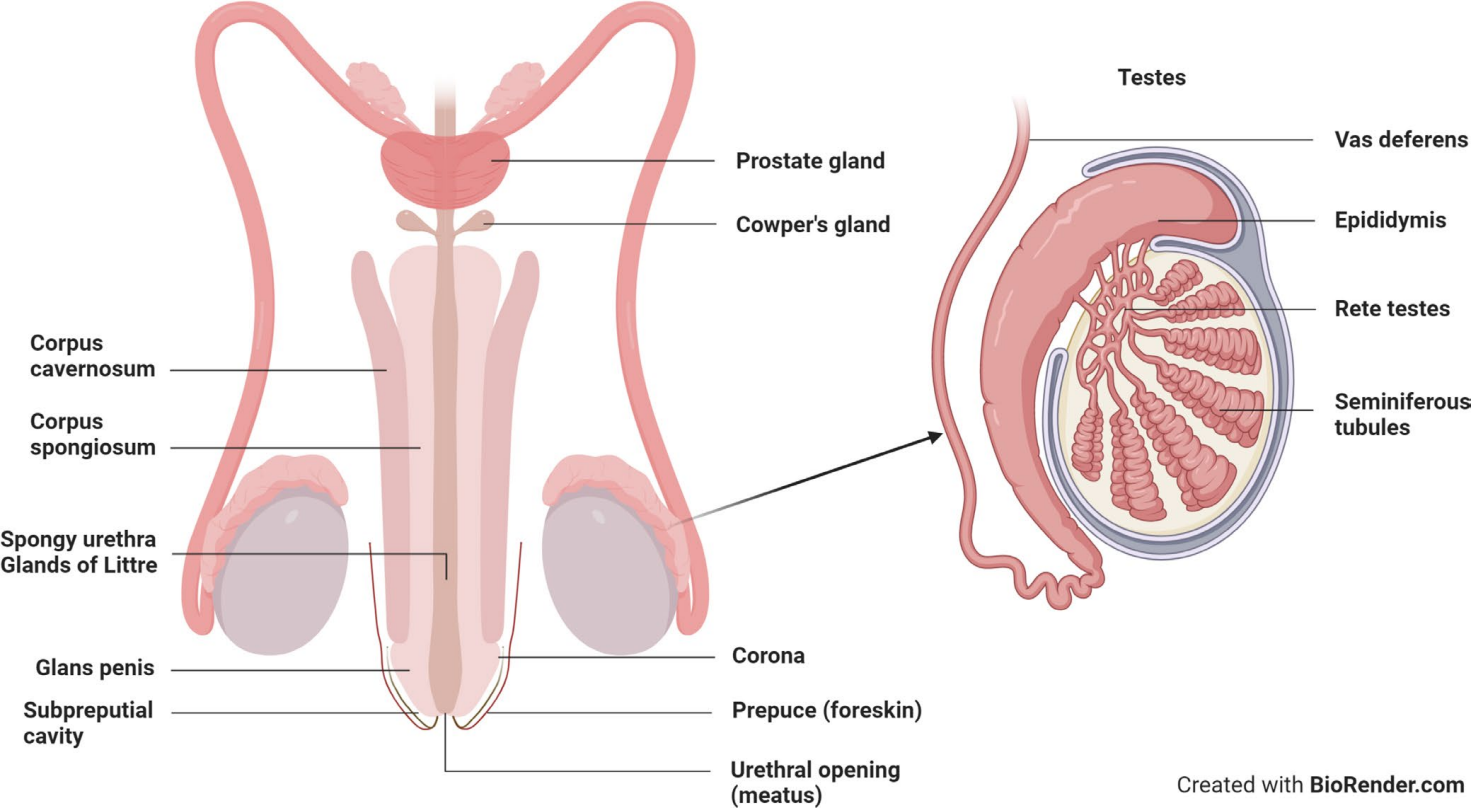
Structure of the male genital tract. The male genital tract is made up of the penile urethra and the testes. The penile urethra is lined with a less resilient non‐keratinised pseudostratified glandular columnar epithelium and is a primary site for infection in men. The testes can be divided into two main regions, the seminiferous tubules and the interstitial spaces between the tubules. The testes are responsible for the production of testosterone and spermatogenesis

Urine and semen are secreted from the penile urethra, which originates at the bladder and is approximately 20 cm in length and 1–2 cm in diameter.^106^, ^117^ In contrast to the foreskin, the urethra is lined with non‐keratinised pseudostratified glandular columnar epithelium, which is less resilient to HIV infection.^117^, ^129^, ^130^ Given that the epithelium of the penile urethra confers reduced protection against HIV entry and contains a high density of intraepithelial immune cells, this serves as a primary site for infection by sexually transmitted infections (STIs), including HIV.^106^, ^107^, ^117^, ^130^, ^131^, ^132^, ^133^ The epithelium of the urethra also contains several deep invaginations called the periurethral glands of Littre.^117^ These Littre glands are responsible for pre‐ejaculate secretion that neutralises residual urine in the urethral lumen and acts as lubrication during condomless sex.^117^

The testes can be divided into two main regions; these are the interstitial spaces between the tubules and the seminiferous tubules.^131^, ^134^ The testes are responsible for the production of testosterone^134^, ^135^ and spermatogenesis, which occurs in the coiled seminiferous tubules.^136^, ^137^, ^138^ The seminiferous tubules connect to the head of the epididymis and then to the vas deferens via the rete testes.^137^ The seminiferous tubules are made up of Sertoli cells that surround the spermatogenic cells and provide essential nutrients to the spermatozoa.^134^, ^135^ The peritubular myoid cells are smooth muscle cells that surround the seminiferous tubules of the testis and provide structural integrity to the tubules.^137^ Peritubular myoid cells are contractile cells that are involved in the maturation and transport of the spermatozoa into the epididymis.^139^ Leydig cells are adjacent to the seminiferous tubules and are the most abundant cells within the interstitial space. These cells are responsible for the production of testosterone and small amounts of oestradiol which facilitate the development of spermatozoa.^137^

## 5 SEMEN COMPOSITION AND IMPLICATIONS FOR HIV INFECTION

Semen contains a mixture of spermatozoa, seminal plasma (SP), microbes and several bioactive molecules known to both promote and inhibit female genital inflammation. Semen contains secretions from the prostate gland and seminal vesicles.^137^ These secretions contain high levels of E‐series prostaglandins (PGE) and transforming growth factor (TGF)‐β, which are known to have potent immunomodulatory effects.^12^, ^16^, ^29^, ^30^, ^31^, ^140^ TGF‐β and PGE2 in semen are commonly associated with anti‐inflammatory properties, including suppressing neutrophils, natural killer cells and dendritic cells (DCs).^29^, ^141^, ^142^ However, in cervical biopsies, PGE2 was shown to stimulate the production of the chemotactic cytokine interleukin (IL)‐8 and inhibit the production of the secretory leukocyte peptidase inhibitor, an enzyme with anti‐HIV activity.^15^ Semen also contains several other cytokines (including IL‐1α, IL‐1β, IL‐2, IL‐7, IL‐8, IL‐10, IL‐15, IL‐17, granulocyte‐macrophage colony‐stimulating factor [GM‐CSF], granulocyte colony‐stimulating factor [G‐CSF], monocyte chemoattractant protein (MCP)‐1, MIP‐1α, MIP‐1β, regulated on activation, normal T cell expressed and secreted [RANTES], fibroblast growth factor [FGF]‐2, growth‐related oncogene [GRO]‐α, tumour necrosis factor [TNF], vascular endothelial growth factor [VEGF], and fractalkine), hormones, immunoglobulins and other proteins.^10^, ^13^, ^17^, ^18^, ^19^, ^20^, ^143^, ^144^ These semen‐derived cytokines are involved in immune cell recruitment and the maturation and proliferation of monocytes, T cells, B cells, DCs and natural killer cells.^145^, ^146^, ^147^ Semen contains high levels of IL‐7, which at similar concentrations in cervicovaginal and lymphoid tissues were shown to enhance HIV‐1 replication and prevent apoptosis of CD4+ T cells.^19^, ^148^ Additionally, semen contains endogenously produced lymphocytes including CD4+ and CD8+ T cells.^149^ Semen also harbours a diverse array of microbes derived from the penile urethra and upper MGT.^24^, ^25^, ^26^ The most abundant bacterial taxa in semen include among others *Streptococcus*, *Staphylococcus*, *Corynebacterium*, *Lactobacillus*, *Prevotella*, *Anaerococcus*, *Finegoldia*, etc.^24^, ^25^, ^26^ Additionally, protein deposits known as amyloid fibrils have also been identified in semen, their physiological function is to mediate the selection and clearance of damaged sperm.^150^ However, these semen‐derived amyloid fibrils also greatly enhance HIV infection by facilitating the binding of HIV virions to their cellular targets for infection.^151^, ^152^, ^153^, ^154^, ^155^ Importantly, semen composition may be altered in the presence of HIV and other STIs resulting in an increased pro‐inflammatory immune response at the FGT, which may further impact HIV susceptibility in women.^18^, ^156^, ^157^, ^158^, ^159^, ^160^, ^161^

## 6 CONTRIBUTIONS OF SEMEN TO FEMALE GENITAL INFLAMMATION

### 6.1 Impact of semen exposure on cytokine biomarkers of FGT inflammation

**FIGURE 2:**
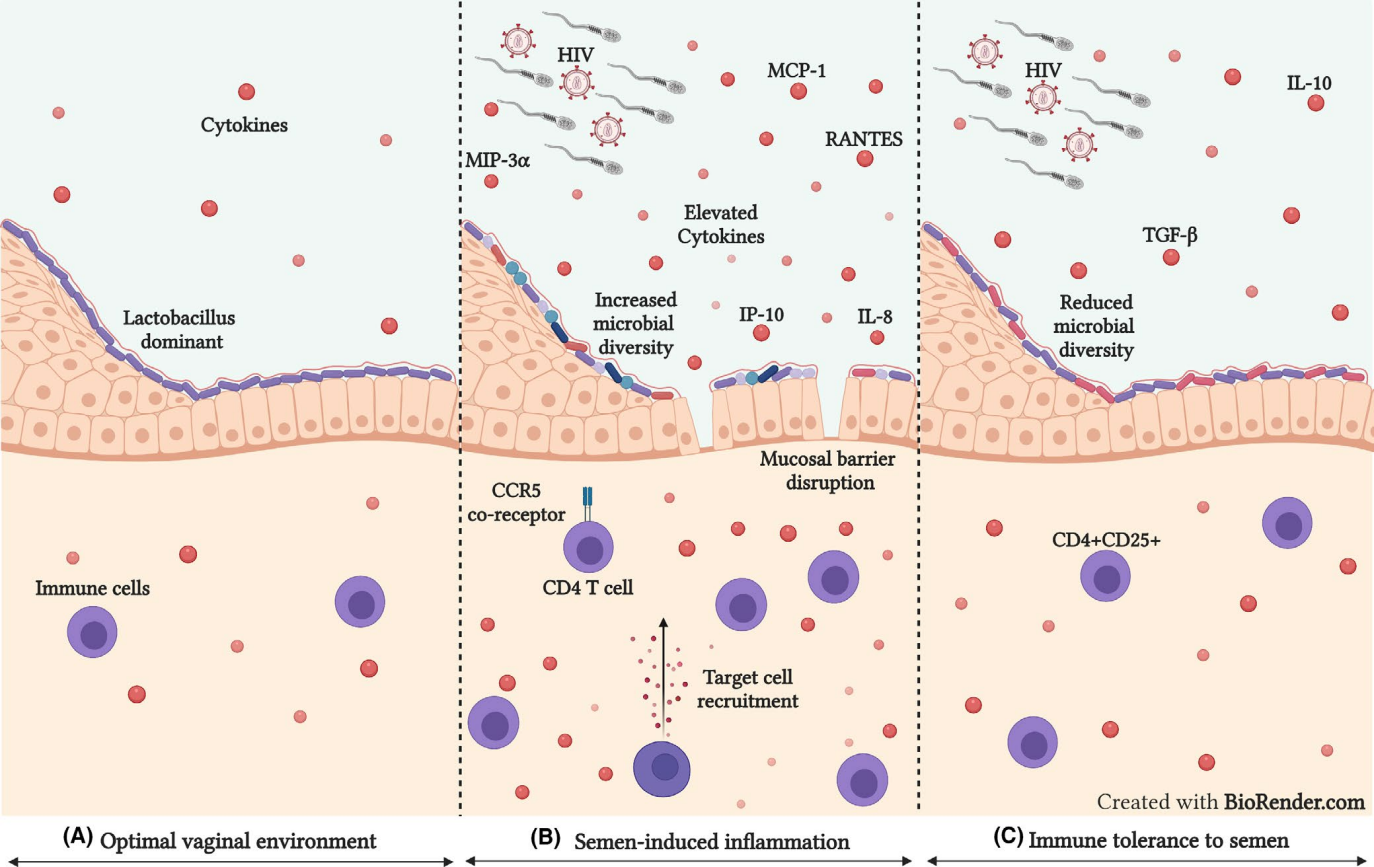
Alterations at the female genital mucosa in response to semen. (A) An optimal vaginal environment contains few cytokines and immune cells. The vaginal microbiome is dominated by Lactobacillus spp. and the mucosal barrier does not contain microabrasions. (B) The pro‐inflammatory components in semen induce cytokine production and target cell recruitment to the FGT. Semen and condomless sex may induce microabrasions in the epithelial barrier and alterations in the vaginal microbiome. (C) The anti‐inflammatory components of semen, including TGF‐β and IL‐10, are associated with fewer cervicovaginal cytokines and expansion of the Treg immune cell (CD4+CD25+) population. Additionally, since homeostasis of the vaginal microbiome is quickly restored after exposure to semen, a tolerogenic immune response to semen may be associated with minor changes in the vaginal microbiome

The immunomodulatory components of semen induce alterations at the FGT to facilitate conception but may also contribute to genital inflammation and HIV risk in women (Figure 2).^14^, ^18^, ^19^, ^22^, ^150^, ^153^ Exposure to semen and SP is associated with short‐term alterations in several cytokines (including IL‐1α, IL‐6, IL‐8, IL‐12p70, TNF‐α, TNF‐β, IP‐10, leukaemia inhibitory factor [LIF], MCP‐1, MCP‐3, RANTES, GM‐CSF, G‐CSF, GRO‐α, MIP‐3α, VEGF, FGF‐2 and fractalkine) at the lower and upper FGT.^10^, ^11^, ^13^, ^20^, ^21^, ^22^, ^162^, ^163^, ^164^, ^165^ Of particular importance is IL‐1α, IL‐6, IL‐8, TNF‐α, MIP‐3α, MCP‐1, RANTES and IP‐10, which have been used to define female genital inflammation.^2^, ^3^ The β‐chemokines MIP‐1α, MIP‐1β and RANTES are CCR5 ligands that recruit HIV target cells to the FGT but also competitively bind to the CCR5 co‐receptor.^93^ Vaginal epithelial cells previously exposed to semen had elevated concentrations of MIP‐3α (CCL20), a chemokine involved in the recruitment of Langerhans cells to the epithelium.^163^ MIP‐3α induces chemotaxis of CCR6+ cells, including Th17 cells, the preferential targets for HIV infection,^90^, ^166^, ^167^ and may therefore increase the availability of HIV susceptible cells at the female genital mucosa. However, in addition to its chemoattractant properties, MIP‐3α also exhibits anti‐HIV activity through competitive binding to the CCR6 receptor.^90^, ^168^ Sharkey et al^11^ demonstrated that exposure to semen induced the expression of IL‐1β, IL‐6 and LIF by endometrial epithelial cells. Expression of these cytokines triggers the recruitment and activation of macrophages, DCs and neutrophils.^11^ Similarly, a study conducted on SP‐treated endometrial epithelial cells and stromal fibroblasts demonstrated an upregulation of several cytokines.^20^ The presence of semen in the female genital mucosa upregulates the production of pro‐inflammatory and chemotactic cytokines,^10^, ^11^, ^13^, ^20^, ^21^, ^22^, ^162^, ^163^, ^164^, ^165^ with several of these associated with leukocyte recruitment and reduced mucosal barrier integrity,^2^, ^3^ both significant contributors to the ability of HIV to penetrate and access target cells at the FGT.

### 6.2 Impact of semen on immune cells at the female genital mucosa

Since semen is initially recognised as foreign in the FGT an immune response is mounted, resulting in cytokine upregulation and the chemotaxis of immune cells. In reproduction, this pro‐inflammatory immune response is necessary for the removal of excess and abnormal sperm.^29^, ^169^ However, these semen‐induced alterations may also increase susceptibility to HIV infection in women. Semen‐derived PGE2 has been associated with the recruitment and activation of HIV target cells.^162^, ^170^ PGE2 in SP was shown to induce prostaglandin‐endoperoxidase synthase‐2 (PTGS2) expression in the cervix of women, where it regulates the tolerogenic phenotypes of DCs and macrophages in the postcoital inflammatory response.^11^, ^16^ The expression of PTGS2 in vaginal cells is also related to an increased susceptibility to HIV and other STIs.^162^ Recent condomless sex has been associated with an influx of CD14+ macrophages, CD1a+ dendritic cells and CD8+ T cells to the cervical epithelium and stroma.^11^ Additionally, SP treatment significantly induced chemotaxis of CD14+ monocytes and CD4+ T cells in endometrial epithelial cells and stromal fibroblasts.^20^ SP also upregulates the expression of the HIV co‐receptor CCR5+ on CD4+ T cells and *in vitro* in HeLa cells.^171^, ^172^ Similarly, we have recently demonstrated that higher cervicovaginal Y‐chromosome DNA (YcDNA) concentrations and prostate‐specific antigen (PSA) detection, both indicative of recent semen exposure, are associated with increased frequencies of activated CD4 + HLA‐DR + T cells and CD4 + CCR5 + HLA‐DR + HIV targets, respectively (Jewanraj et al, 2021; accepted).

A Treg immune response is induced soon after semen exposure since prolonged inflammation at the FGT may reduce the odds of fertilisation and pregnancy.^12^, ^16^, ^30^, ^31^, ^173^ Semen‐derived TGF‐β and PGE induce a shift from an initial Th1 to a Th2 immune response by promoting Treg cell differentiation and expansion.^12^, ^29^, ^30^ The induction of a Treg immune response results in tolerance of the paternal alloantigen at the time of embryo implantation.^12^, ^31^, ^32^ Prostaglandins in semen may also upregulate the production of the anti‐inflammatory cytokine IL‐10.^137^ Consistent with this, we and others have demonstrated elevated cervicovaginal IL‐10 concentrations in response to recent semen exposure.^13^, ^165^ Additionally, prostaglandins prevent an immune response at the FGT by inhibiting macrophage cytokine production and T‐cell proliferation.^29^, ^30^, ^169^, ^174^, ^175^ Although this induction of immune tolerance may be protective for the paternal alloantigen, this dampened immune response may prevent pathogen clearance at the female genital mucosa.

In addition, studies have demonstrated that prior and prolonged exposure to the same donor's semen improved fertility and reduced preeclampsia rates in women, highlighting the importance of immune tolerance to semen in these contexts.^176^, ^177^, ^178^, ^179^, ^180^ Furthermore, a recent study conducted in rhesus macaques demonstrated that repeated vaginal exposure to semen resulted in lower CCR5 expression on CD4+ T cells and reduced infection by Simian Immunodeficiency Virus.^181^ These findings suggest that semen exposure to new or multiple concurrent partners may induce a greater and prolonged inflammatory response, which is associated with adverse pregnancy outcomes and possibly an increased risk of HIV transmission.^176^, ^177^, ^178^, ^180^, ^181^ Immune tolerance may be lost on exposure to semen from a new partner, resulting in a more pronounced immune response and suggests a biological link for the relationship between partner concurrency and HIV risk in South African women.^182^

### 6.3 Impact of semen exposure on the vaginal microbiota

Bacterial vaginosis is a state characterised by a shift in the vaginal microbiome from Lactobacillus dominance to a more diverse spectrum of facultative anaerobes.^62^, ^183^ Condomless sex has been associated with BV occurrence,^28^, ^184^, ^185^, ^186^ and increases in *Escherichia coli* at the FGT.^185^, ^187^, ^188^, ^189^ Semen contains a diverse array of bacteria that are introduced into the vagina during condomless sex.^24^, ^25^, ^26^ Additionally, the MGT itself (including the penile skin, meatus, glans/corona and the subpreputial cavity) also contains a diverse array of bacterial taxa that may be transferred to the FGT in the absence of ejaculation and semen exposure.^24^, ^25^, ^26^, ^27^, ^28^, ^187^, ^190^, ^191^ A high level of concordance has been observed between the MGT microbiome composition and BV incidence in female partners.^27^, ^28^, ^190^ In addition, semen has an alkaline pH range between 7.2 and 7.8, capable of buffering the acidic pH of vaginal fluid.^23^, ^192^, ^193^ This neutralisation of the vaginal pH may promote a shift in the vaginal microbiome to a BV‐associated state that is conducive to HIV‐1 infection.^4^, ^5^, ^7^, ^52^, ^69^, ^193^, ^194^ Several factors in semen may also inhibit the activity of extracellular H_2_O_2_ produced by Lactobacilli species and thus promote the growth of BV‐associated microbes.^195^ We have demonstrated that recent semen exposure is associated with increased detection of BVAB‐2, *Prevotella bivia*, and *G. vaginalis* and reduced detection of *Lactobacillus jensenii* in vaginal specimens (Jewanraj et al, 2021; accepted).^165^ Increases in other gut‐associated microbes have also been observed in the FGT after protected sexual intercourse, suggesting that these alterations in the vaginal microbiota may also be associated with mechanical contamination rather than just semen itself.^185^, ^187^ These studies suggest that semen exposure and sexual intercourse may promote a shift in the microbial environments of the FGT that may facilitate HIV infection in women.^4^, ^5^, ^7^, ^165^

### 6.4 Impact of sexual intercourse and semen exposure on the vaginal epithelial barrier

An intact vaginal epithelial barrier is the primary host defence against HIV entry and infection. Reduced epithelial barrier integrity may facilitate HIV access to target cells at the FGT. Colposcopic examination of the vaginal mucosa revealed that friction during consensual sexual intercourse might cause microabrasions in the epithelial barrier.^196^, ^197^, ^198^ Additionally, pro‐inflammatory cytokines within semen may also increase the permeability of the vaginal epithelium. Interferon‐gamma in semen may increase epithelial permeability by inducing macropinocytosis of tight junction proteins.^199^ Semen‐derived IL‐1β may also increase vaginal epithelium tight junction permeability through the activation of the NF‐κβ pathway.^200^ Elevated levels of MMPs have also been linked to reduced mucosal barrier integrity, increased cervicovaginal cytokine production, immune cell recruitment at the vaginal mucosa and increased HIV transmigration.^3^, ^6^ We have recently demonstrated that semen exposure is associated with increased concentrations of MMP‐2 and their inhibitors in vaginal specimens.^165^ An increased HIV incidence has been observed among women with compromised epithelial barrier integrity through the enhanced ability of HIV‐1 to penetrate the vaginal epithelium.^11^, ^96^, ^97^, ^98^, ^201^

## 7 THE ROLE OF SEXUAL INTERCOURSE AND SEMEN EXPOSURE ON TOPICAL PrEP EFFICACY

In addition to its role in female genital inflammation and immune activation, semen exposure and sexual intercourse may also undermine topical pre‐exposure prophylaxis (PrEP) efficacy^202^, ^203^, ^204^ and has additional implications for HIV susceptibility in women. The physiological changes that occur during coitus may alter PrEP efficacy by changing the surface area of the vagina and redistributing cervicovaginal fluid and topically applied microbicides.^205^, ^206^ In clinical trials, vaginal microbicide gels PRO 2000 and cellulose sulphate failed to confer protection against HIV‐1 transmission in women.^207^, ^208^ In vitro assays demonstrated a significant reduction in the antiviral activity of PRO 2000 gel following sexual intercourse.^204^ Tenofovir gel concentrations were also significantly reduced in cervicovaginal lavage and vaginal and cervical tissues after coitus.^203^ These findings were likely due to the redistribution of the microbicide gels in the vagina during sexual intercourse.

Semen and SP itself contains several bioactive molecules and may also alter the antiviral activity of microbicides.^202^, ^204^, ^209^, ^210^ SP was shown to interfere with the HIV‐1 and herpes simplex virus (HSV)‐2 inhibitory activity of PRO 2000 and cellulose sulphate microbicides.^202^, ^209^, ^210^ Seminal proteins, fibronectin and lactoferrin competitively inhibited the binding of the microbicides to their target on the HSV envelope.^210^ The reduced antiviral activity of these microbicides may also be due to electrostatic interactions between cationic SP polyamines and the polyanions of the microbicides.^204^, ^209^, ^210^, ^211^ Zirafi et al^202^ demonstrated that seminal amyloids enhance HIV infection and also contribute to the reduced antiviral activity of microbicides. Additionally, we previously demonstrated that recent semen exposure was associated with increased detection of *G. vaginalis* and biomarkers of inflammation in vaginal specimens (Jewanraj et al, 2021; accepted), both of which contribute to diminished topical PrEP efficacy in women.^212^, ^213^ These studies suggest that sexual intercourse and semen itself may also reduce the efficacy of topical PrEP in women and highlights the need to assess and control for these factors.

## 8 BIOMARKERS OF SEMEN EXPOSURE

Research primarily relies on self‐reports of condom use and sexual behaviour, which may lead to inaccurate data interpretation due to reporting bias.^214^, ^215^, ^216^, ^217^, ^218^ Although biomarkers of semen exposure were developed for use in forensics, they also have several useful applications in HIV prevention research. Semen biomarkers can be used to control for semen‐induced alterations at the FGT, assess condom use in clinical trials and determine the efficacy of barrier contraceptives and microbicides.^165^, ^219^, ^220^, ^221^, ^222^, ^223^, ^224^, ^225^, ^226^, ^227^, ^228^, ^229^ Biomarkers that have been previously used to detect semen in vaginal specimens include PSA, YcDNA, semenogelins, acid phosphatase and sperm detection by microscopy.^165^, ^226^, ^227^, ^228^, ^229^, ^230^, ^231^, ^232^, ^233^, ^234^ PSA and YcDNA detection are the most well‐studied and commonly used biomarkers of semen exposure.^235^ PSA is present in high concentrations in semen, and detection in vaginal fluid usually indicates semen exposure within 48 h.^226^, ^236^, ^237^, ^238^, ^239^ We and others have demonstrated that PSA detection in vaginal specimens, a proxy for recent semen exposure, is associated with a pro‐inflammatory immune response at the FGT (Jewanraj et al, 2021; accepted).^227^, ^229^ Conversely, YcDNA is a more stable biomarker and is detectable in vaginal specimens up to 15 days after coitus.^219^, ^231^, ^235^, ^240^ Since YcDNA is detectable in the presence of spermatozoa, it is an ideal measure of the probability of pregnancy.^219^ These semen biomarkers may be suitable for different studies depending on the residence time of the biomarker and the study outcome, such as the probability of pregnancy, infection or genital inflammation. Routine objective screening for semen biomarkers may avoid the discrepancies associated with self‐reported data and may lead to more reproducible study outcomes. Additionally, given the immunomodulatory properties of semen, these biomarkers can be used to control for semen's impact on the immune and microbial microenvironments of the FGT.

## 9 CONCLUSION

Identifying factors associated with female genital inflammation and limiting their impact on HIV risk is particularly important in high HIV burden areas. Semen is a highly immunomodulatory fluid and is the primary vector for HIV transmission to women during condomless sex. However, most studies of mucosal immunity are conducted in the absence of semen or without consideration of its immune impact on the female genital mucosa. Semen exposure is associated with a short‐term inflammatory response at the FGT which is quickly resolved to facilitate immune tolerance to the paternal antigens. Albeit short‐lived, a semen‐induced pro‐inflammatory immune response may promote genital inflammation and HIV risk in women. Additionally, semen and condomless sex may also modulate topical PrEP efficacy and have additional implications for HIV risk in women. Future clinical and immunological studies of HIV and other STIs should consider semen's contribution to the immune and microbial environments of the FGT. We suggest that STI/HIV research may benefit from routine screening for semen biomarkers in vaginal specimens to account for its impact on female genital inflammation.

## CONFLICT OF INTEREST

The authors declare no conflicts of interest.

## Data Availability

Data sharing not applicable to this article as no datasets were generated or analysed during the current study.
